# Collapse of Lipid Membranes into Distended Lipidic Cubic Phases at High Solvent Levels, Membrane Remodelling, and Self-Repair

**DOI:** 10.1021/jacs.5c07146

**Published:** 2025-07-01

**Authors:** Vivien Yeh, Alice Goode, Nikul Khunti, Julie Watts, Christopher Parmenter, Michael Fay, David Johnson, Nathan Cowieson, Boyan Bonev

**Affiliations:** † School of Life Sciences, 6123 University of Nottingham, Nottingham NG7 2UH, U.K.; ‡ 120796 Diamond Light Source, Didcot, Oxfordshire OX22 0DE, U.K.; § Nanoscale and Microscale Research Centre, 6123 University of Nottingham, Nottingham NG7 2RD, U.K.; ∥ School of Pharmacy, 6123 University of Nottingham, Nottingham NG7 2RD, U.K.; ⊥ Mitsubishi Chemical Methacrylates, Wilton Centre, Wilton, Redcar TS10 4RF, U.K.

## Abstract

Performance plastics, such as poly­(methyl methacrylate), underpin the modern economy. Global manufacturing of plastic precursors relies on fossil carbon sources, and the urgently needed shift toward renewable carbon use through biofermentation is hindered by the low tolerance of producer strains to methacrylate esters. The principal mode of butyl methacrylate cellular toxicity is membrane disruption. To understand this process, the conditions for membrane stability, and recovery after solvent shock, we investigate the phase stability of hydrated lipid membranes at high levels of a key intermediate, butyl methacrylate. We assess the role of *cis*- vs *trans*-unsaturation in 18-carbon chain phospholipids on butyl methacrylate-induced phase conversion and polymorphism. Using ssNMR, SAXS and cryoEM, we demonstrate the formation of stable lipidic cubic phases in hydrated lipid/solvent (*cis*-chain phospholipid lipid/butyl methacrylate) systems at a 1:6 molar ratio entirely lacking monoolein. Transient lipidic cubic phases form in *trans*-chain phospholipid/butyl methacrylate systems, which slowly convert to bilayers through a spontaneous “membrane healing” process during recovery after solvent shock. The observed bicontinuous nanostructures with a cubic phase architecture coexist with a stable, monocontinuous hydrated phase of the same morphology but with simpler topological connectivity, which demonstrates that phase stability in cubic phases does not require topological complementarity. We propose *trans*-lipid substitutions in membranes of fermentative strains as a key step toward sustainable production of methacrylate esters.

## Graphical abstract

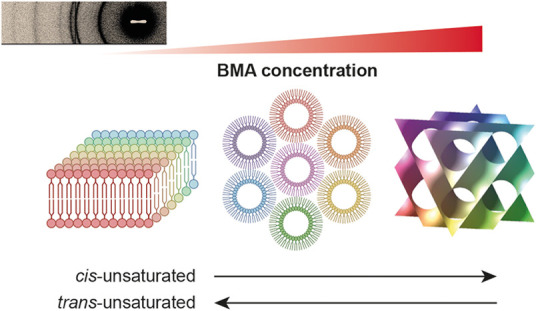

A widely used performance plastic, poly­(methyl methacrylate) (PMMA) has increasing global production estimated at 2.8 MT p.a. and a predicted market share value of $7.5 billion by 2030.^1^ PMMA finds applications in a wide range of applications, such as shatter resistant glass substitute, stable coating polymers and paints, and durable medical and dental applications, to name a few. Production of methyl methacrylate, the monomeric PMMA precursor, relies on the use of petrochemical sources of carbon in the sulfuric acid process, and sustainable technologies that make use of renewable, nonfood carbon are urgently needed. Industrial technologies rely on the biofermentative production of butyl methacrylate (BMA), which is subsequently transesterified to methyl methacrylate.^2^ The commercial viability of this approach is dependent on large-scale continuous fermentation, which is presently limited by product toxicity to the producer strains at levels nearly sufficient for maintaining continuous fermentation.

BMA is a solvent with an intermediate hydrophobicity of logP = 2.9 ^3^ and readily partitions into the hydrophobic interior of lipid membranes. Previous studies have shown high membrane tolerance and stability in the presence of BMA at up to 80 mol % to a membrane lipid,^4^ where a hydrophobic solvent was accommodated well between the lipid chains at solvent-to-lipid molar ratios as high as 4:1. This value is close to the highest levels of BMA tolerated by bacteria (∼0.8 g/L),^5^ which points to membrane collapse as the leading mechanism of BMA toxicity to bacterial cells. The membrane fate following such solvent-driven collapse remains unexplored, and ways to stabilize the cell membranes are urgently needed. Higher tolerance to BMA was observed in bilayers composed of *trans*- vs. *cis*-unsaturated phospholipids.^4^ The benefits of *trans*-unsaturated lipids to solvent tolerance have been suggested in *cis–trans* isomerase-containing fermentation strains of *Pseudomonas putida.* ^6^ It has been shown that tolerance of *E. coli* to various biorenewable products such as alcohol, organic acids and aromatic compounds may be improved through the production of non-native *trans*-unsaturated fatty acids through expression of *cis–trans* isomerase (cti) from *P. aeruginosa.* ^7^

At higher solvent levels, besides a dominant bilayer phase, we have observed the presence of a small fraction of nonbilayer lipid using solid-state NMR (ssNMR),^4^ which can be attributed to lipid cubic phase (LCP) formation. LCPs are bicontinuous macroscopic liquid crystalline phases, commonly observed in monoolein at low hydration levels.^8,9^ They find important applications in membrane protein crystallization and structure determination by X-ray diffraction,^10–13^ pharmaceutical formulations, and food preparations^14^ and serve as a matrix for nanomaterial development.^12,15^ LCP symmetry belongs to the *m*3*m* point group, and generating surfaces for LCPs from reported primitive *Pn*3*m*, gyroid *Ia*3*d*, and diamond *Im*3*m* space groups^16^ are shown in Figure 1.

**Figure 1:**
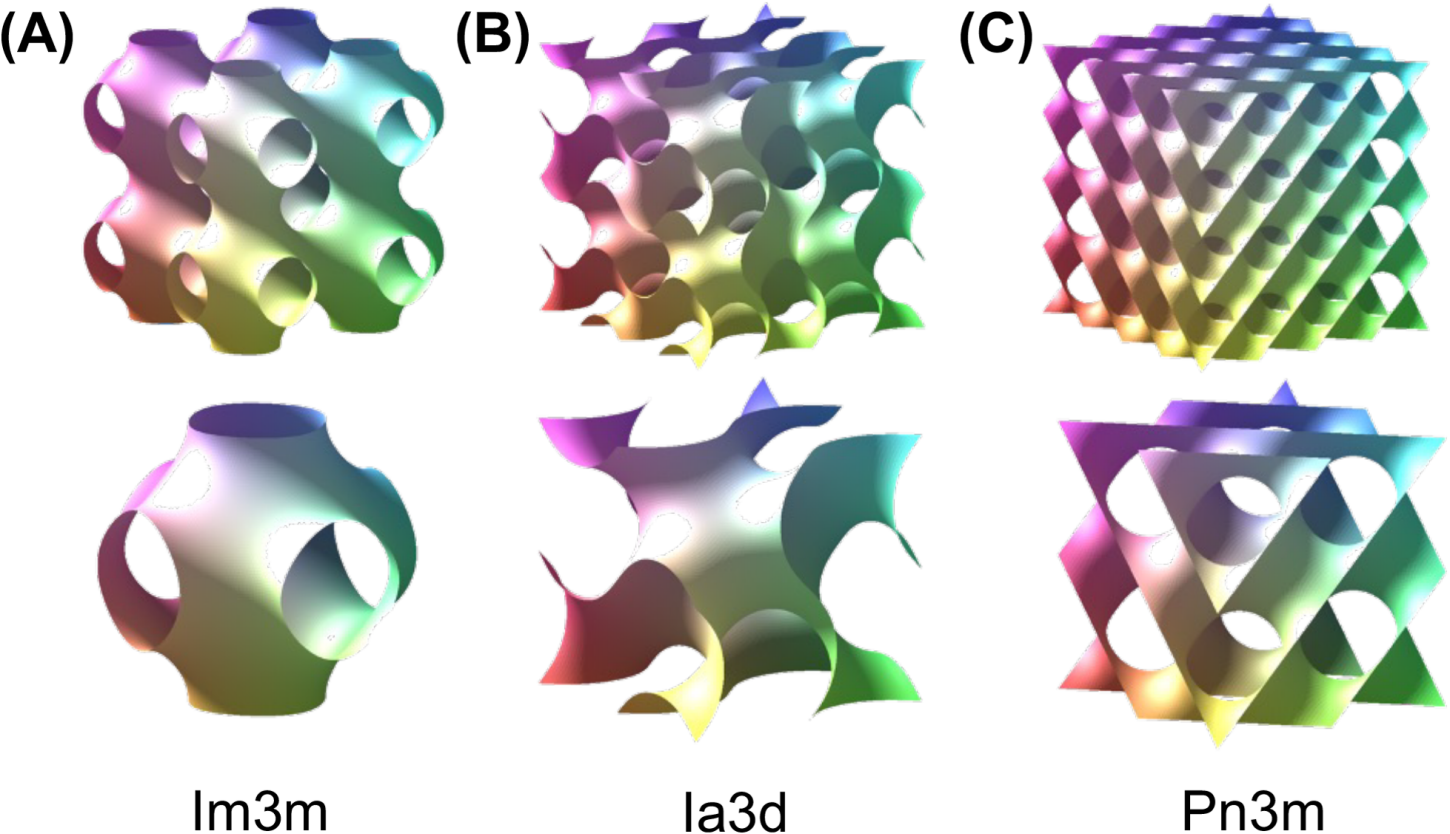
Generating surfaces and topology of LCPs.^17^ Primitive *Im*3*m* (A), gyroid *Ia*3*d* (B), and diamond *Pn*3*m* (C) phases over phase space [−2π, 2π] (top) and [−π, π] (bottom).

Lipid chain composition is a key modulator of the membrane stability. It defines the limits of solvent partitioning into the lipid bilayer as well as the phase stability and tolerance of the bilayer phase. In the presence of very high levels of apolar solvents, lipid membranes can adopt a nonbilayer phase at equilibrium or can repartition back into aqueous emulsions while the bilayer phase is restored.^4,18^

To understand the mechanism of bilayer collapse and BMA toxicity to biological membranes, we prepared hydrated ternary systems composed of hydrated *cis*- or *trans*-unsaturated C18:1Δ9 chain phospholipids in the presence of increasing BMA fractions, in which the BMA molar ratio exceeded the 4:1 membrane stability limit we determined previously.^4^ Here, we report the feasibility of generating distended periodic nanostructures with LCP symmetry in ternary mixtures of phospholipids, BMA, and water. The obtained LCPs from hydrated (C18:1Δ9-*cis* dioleoyl phosphatidyl choline (DOPC)/BMA 1:6 are stable, while LCPs from C18:1Δ9-*trans* dioleoyl phosphatidyl choline (DEPC)/BMA 1:6 undergo a recovery through an H_II_ intermediate to the bilayer phase in a “membrane healing” process.

We hypothesize that membrane stability and polymorphic phase conversion are driven by solvent compatibility with the lipid chain region. To investigate the role of BMA on membrane stability and polymorphic phase state, we use bilayer-forming hydrated monounsaturated DOPC and DEPC models,^4^ which we combine with increasing molar fractions of BMA. Lamellar, inverted hexagonal, and cubic phases are identified through distinct wide-line ^31^P ssNMR intensity distributions^19^ (Figure S1). The symmetry and characteristic dimensions of the periodic lipid phases were obtained from small angle X-ray scattering (SAXS).^20^

Oleic chains are common in bacterial membrane phospholipids.^21^ We illustrate the path of solvent-induced phase conversion during membrane collapse from bilayer to nonbilayer phases in hydrated mixed systems of DOPC with BMA at molar ratios of 2:3, 1:3, 1:6 and 1:9 (Figure 2). The lowest fraction 2:3 DOPC/BMA steers the bilayer into coexistence with an inverted hexagonal H_II_ phase and a contribution from a cubic phase (Figure 2A). The equilibrium is shifted entirely to a dominant H_II_ phase coexisting with an LCP at a DOPC/BMA ratio of 1:3 (Figure 2B). Bragg reflections corresponding to these phases (Figure 2C and D) corroborate the ^31^P NMR observations (Figure 2A and B, respectively).

**Figure 2:**
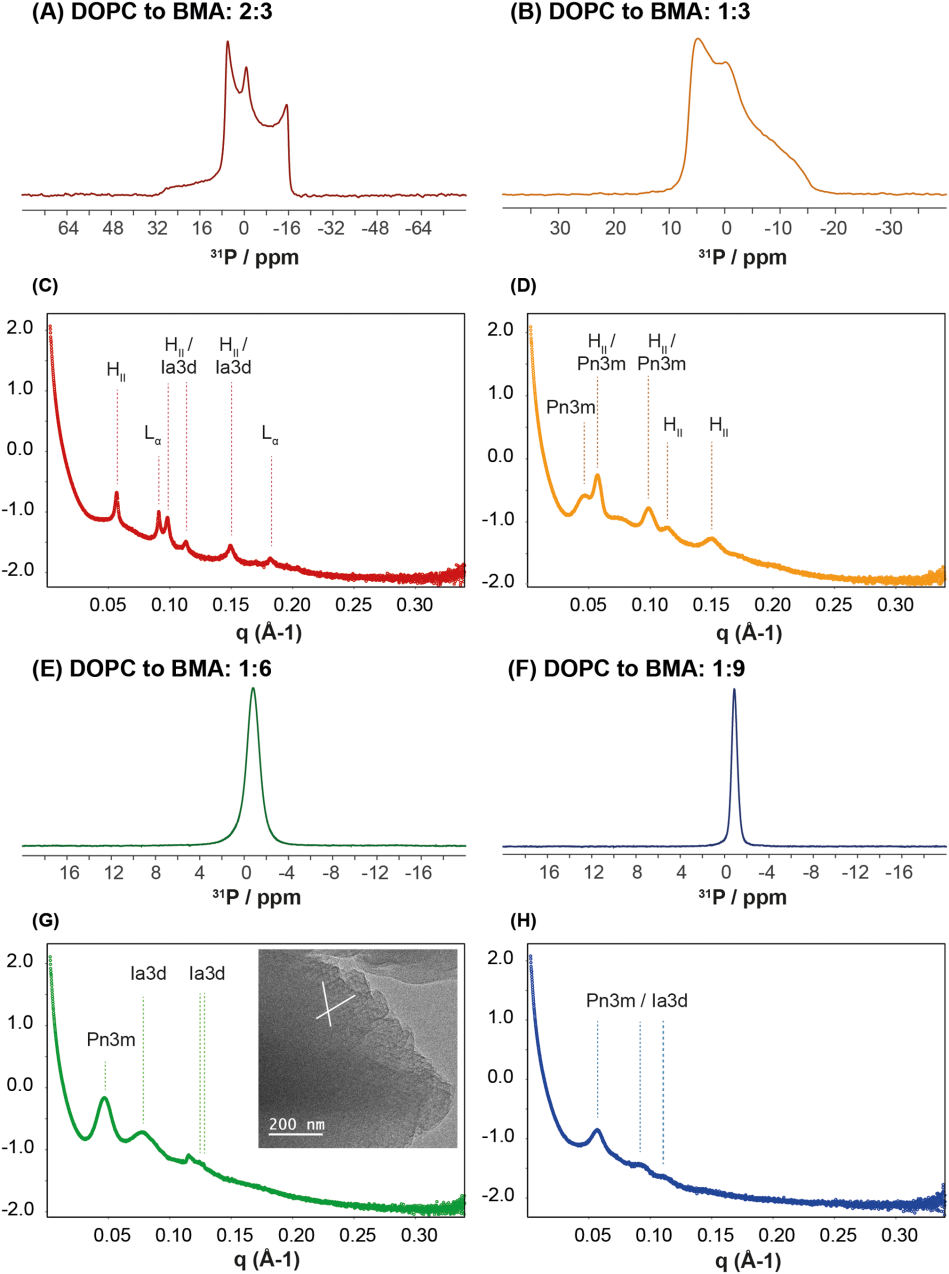
BMA molar fraction dictates the phase polymorphic conversions in hydrated DOPC ternary systems. Wideline ^31^P ssNMR spectra (A, B, E, F) and 1D SAXS diffraction patterns (C, D, G, H) from hydrated DOPC/BMA at molar ratios of 2:3 (A, C, red), 1:3 (B, D, yellow), 1:6 (C, E, green), and 1:9 (F, H, blue) at 20 °C. Reflection and phase assignment are detailed in Table S1 and Figure S2.

Wideline ^31^P NMR spectra from hydrated DOPC/BMA 2:3 systems (Figure 2A) show overlapping contributions from a bilayer, an H_II_ phase, and a cubic phase (Figure S1). To understand the structural characteristics of the three coexisting phases in hydrated DOPC/BMA 2:3 mixtures, we used SAXS (Figure 2C). We assign the Bragg reflections, arising from the intrinsic periodicity of the three phases, using a sliding template of *hkl* selection criteria against the experimental diffraction patterns (Table S1) following assignment of SAXS powder diffraction reflections from lipid phases.^16,20^ We observe the coexistence of lamellar phase with repeat *d* of 69.0 Å, slightly swollen compared to pure hydrated DOPC repeat of 62.8 Å^4^, an H_II_ phase with unit cell dimension *a* = 128 Å and the presence of a distended *Ia*3*d* LCP with a unit cell size *a* = 64.1 Å. We estimate the size of the LCP nanocrystals to be on the order of 4200 Å.

At a higher DOPC/BMA ratio of 1:3, the 1D SAXS patterns show the coexistence of H_II_ and cubic phases (Figure 2D). The bilayer phase was completely destroyed, and the powder pattern was absent from the ^31^P NMR spectrum (Figure 2B). The ^31^P NMR line shape was dominated by an inverted powder, revealing the system was primarily in an H_II_ phase, with a coexistent LCP (Figure S1). The Bragg reflections revealed a H_II_ phase with *a* = 128 Å, a *Pn*3*m* phase with unit cell size *a* = 135 Å, and an *Ia*3*d* phase with *a* = 64 Å.

Increasing the BMA concentration to 1:6 DOPC/BMA, both ^31^P wide-line NMR (Figure 2E) and SAXS (Figure 2G) showed the ternary system adopted a pure *Pn*3*m* LCP with characteristic dimension *a* = 134 Å and estimated nanocrystalline diffracting “cubosomes”^15^ on the order of 900 Å.

At the highest BMA concentration of 1:9 DOPC/BMA, ^31^P wide-line NMR spectrum shows a single cubic phase (Figure 2F). The position of the lead SAXS reflection suggests the presence of an *Ia*3*d* cubic phase with *a* = 111 Å, which reveals a distorted phase compared to reported values in monoolein-based LCPs.^20^

We visualize the lipidic nanostructures using cryoEM from mechanically thinned and flash-frozen or FIB-milled mixtures of 1:6 DOPC/BMA, in which we created electron-transparent thin zones. The cubic repeat of *Pn*3*m* lattice with *a* = 136 Å unit cell is seen at the edge of the thinned zone (Figure 2G, inset). The observed pattern is of a macroscopic phase with *Pn*3*m* organization, unlike the reported *Im*3*m* architecture in monoolein cubosomes^15^ (Figure 1).

Lipid chain *trans*-unsaturation permits better packing of the hydrophobic interior than *cis*-lipids while maintaining membrane fluidity. We have previously reported a higher tolerance for BMA in DEPC membranes than in DOPC membranes.^4^ At a BMA molar ratio of 1:6 DEPC/BMA, membrane collapse leads to the formation of a cubic phase, seen as a single isotropic resonance in ^31^P NMR wide-line spectra (Figure 3A). Diffraction patterns from hydrated 1:6 DEPC/BMA systems show Bragg reflections we attribute to coexistence of *Pn*3*m* and *Ia*3*d* phases with *a* = 146 and 107 Å, respectively (Figure 3B), and a nanocrystalline size on the order of 80 nm. In coexistence with the LCPs, we observe a bilayer component with lamellar repeat *d* = 61.6 Å, slightly lower than the lamellar repeat of 64.1 Å observed in the hydrated DEPC membrane due to increased lipid chain disorder in the presence of solvent.

**Figure 3:**
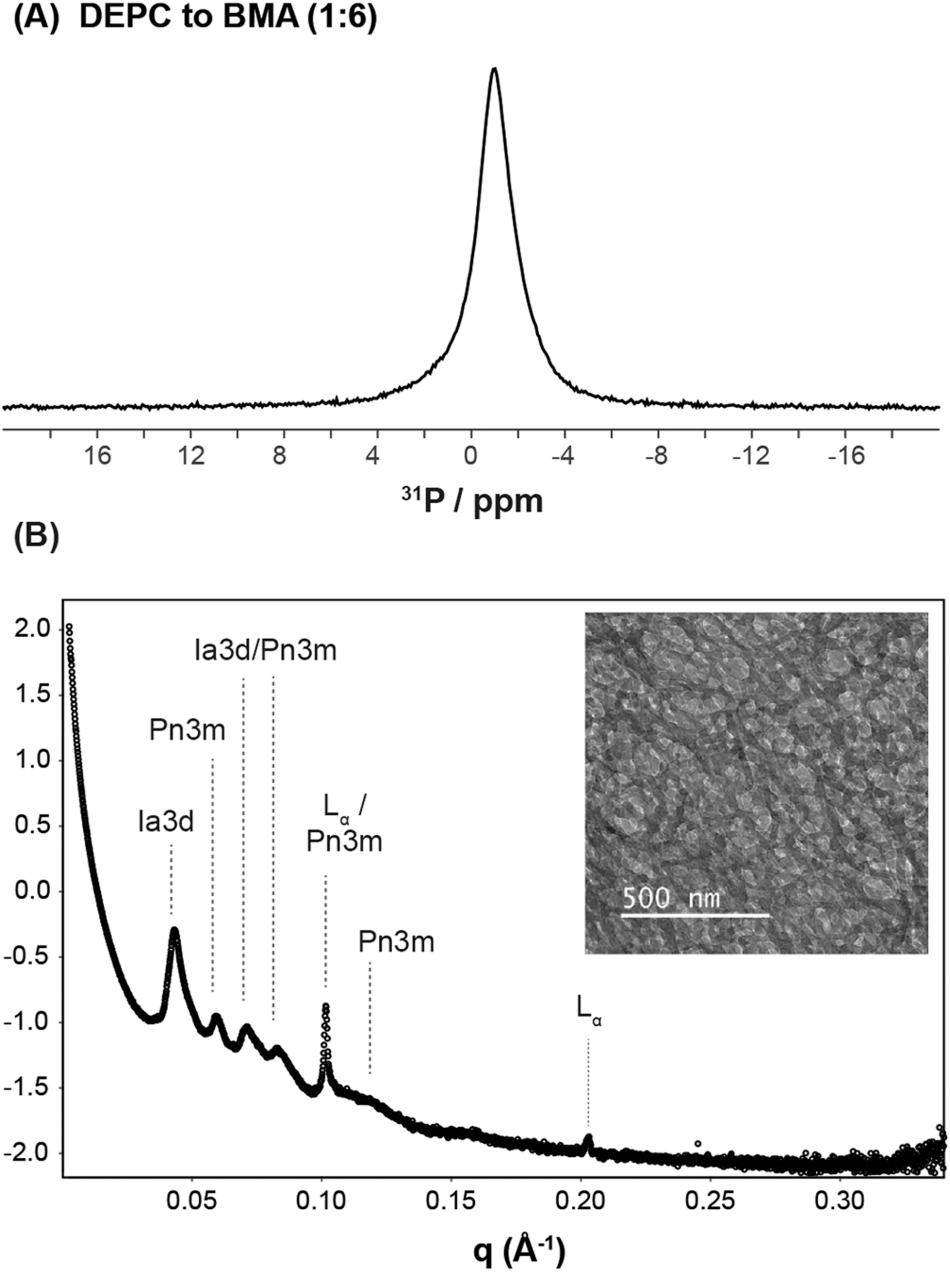
Lipid-phase coexistence in hydrated 1:6 DEPC/BMA ternary systems. Wideline ^31^P ssNMR shows a cubic phase (A) confirmed by SAXS diffraction from LCP with a lamellar contribution (B).

We visualize hydrated DEPC/BMA 1:6 systems using cryoEM from mechanically thinned samples, flash-frozen after thinning. The overall appearance of the ternary phase resembles the reticular structure of a hydrogel, in which we resolve lipid necks and nodes, morphologically characteristic of distended diamond *Pn*3*m* organization (Figure 3B, inset). The structure contains kinetically trapped ordered diffracting nanocrystallites interspersed in the hydrogel matrix of similar morphology with local *Pn*3*m* or H_II_ characteristics. We observe expanded networks of LCP necks and nodes in a stable, standalone monocontinuous assembly without the structural support of a complementary monocontinuous subphase. This shows that bicontinuous complementarity, normally observed in LCPs, is not required for structural stability of LCPs with saddle point curvature morphology.

We use wide-line ^31^P ssNMR to assess stability in hydrated mixtures of DOPC/BMA 1:6 and DEPC/BMA 1:6 over a nine-day period (Figure 4). On day 1, we observed the self-assembly of the DOPC/BMA 1:6 ternary system into a cubic phase (Figure 2E and G), which showed consistent spectral features and remained stable over the entire nine-day period of observation (Figure 4A). By contrast, the DEPC/BMA 1:6 ternary system began the same journey in a mixed phase state (Figure 3A and B), but underwent phase evolution through the increased content of an H_II_ intermediate phase over days 1 and 2 into a bilayer state (Figure 4B). By day 9, all remains of the initially dominant LCP and intermediate H_II_ phases disappeared and the DEPC/BMA 1:6 mixture reached a stable bilayer state (Figure S3), as in DEPC/BMA 1:4 bilayers reported previously.^4^ This observation shows that *trans*-unsaturated DEPC attains a stable bilayer state that retains in the bilayer interior only saturation levels of BMA and is able to “heal” from the LCP state back to the bilayer through an H_II_ intermediate. This remarkable transformation backtraces the mesophase evolution that we observed on increasing BMA levels (Figure 2) and results in stable, fluid bilayer structures.

**Figure 4:**
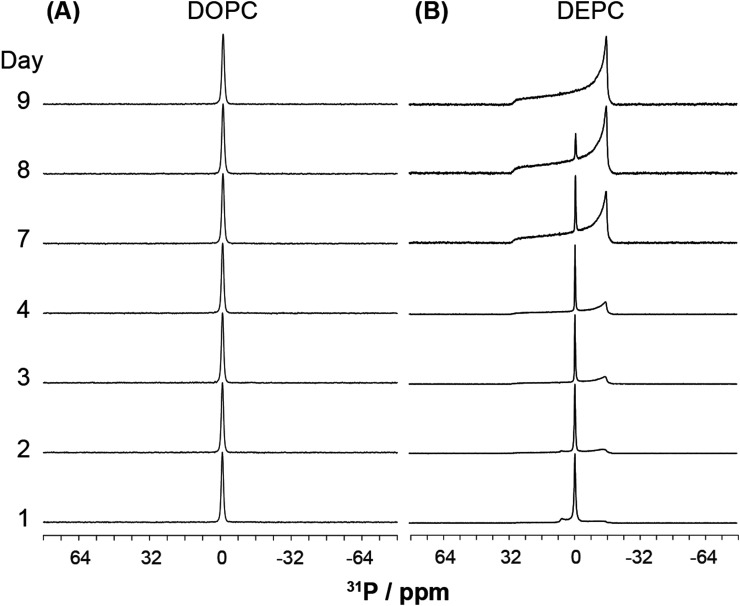
Phase evolution in DOPC (A) and DEPC (B) with a 1:6 lipid/BMA molar ratio at 20 °C. Wideline ^31^P ssNMR spectra recorded over 10 days reveal a stable LCP in DOPC/BMA, while DEPC/BMA phase coexistence underwent conversion to a bilayer phase by day 9.

In this study, we report the formation of LCPs in hydrated phospholipid systems at high levels of solvent, the leading role of chain stereochemistry in phase stability, and the process of membrane recovery from solvent stress in *trans*-unsaturated lipid mixtures. We observe the existence of stable lipidic cubic phases in hydrated *cis*-unsaturated DOPC/BMA ternary mixtures above a 1:6 molar ratio. In the presence of very high solvent titers, DEPC membranes collapse into mixed LCP/H_II_ states, which recover over time to restore the bilayer state. We propose a mechanism of membrane collapse, in which high levels of solvent induce negative curvature from within the bilayer and drive onset of the H_II_ phase and, at higher BMA levels, a conversion to cubic phases. We conclude that hydrated DEPC/BMA mixtures can form macroscopic monocontinuous networks, characteristic of primitive LCPs, which remain topologically stable in the absence of a second, complementary network, commonly associated with LCPs. Membrane stability in fermentative manufacture of solvents can be enhanced through genomic integration of a *cis*–*trans* isomerase^6^ or through directed evolution under solvent selection.^22^

## Supplementary Material



## Notes

Additional experimental details, materials and methods, including SAXS reflection assignments and analysis (PDF)

## Abbreviations

PMMA: poly­(methyl methacrylate)
DOPC: C18Δ9-*cis* dioleoylphosphatidyl choline
DEPC: C18Δ9-*trans* dipalmitoylphosphatidyl choline
MO: monoolein
LCP: lipidic cubic phase
BMA: butyl methacrylate
NMR: nuclear magnetic resonance
ssNMR: solid-state NMR
SAXS: small-angle X-ray scattering
cryoEM: cryo-electron microscopy
FIB: focused ion beam
