# Supplementary material for: Collapse of Lipid Membranes into Distended Lipidic Cubic Phases at High Solvent Levels, Membrane Remodelling, and Self-Repair
Source: J Am Chem Soc. 2025 Jul 1;147(28):24235–40. doi: 10.1021/jacs.5c07146 (PMC12272544; doi:10.1021/jacs.5c07146)
Supplement: Supplementary file 1 [file ja5c07146_si_001.pdf]

# Collapse of lipid membranes into distended lipidic cubic phases at high solvent levels, membrane remodelling and self-repair – Supplement

**By:**

Vivien Yeh[1], Alice Goode[1], Nikul Khunti[2], Julie Watts[3,4], Christopher Parmenter[3], Michael Fay[3], David Johnson[5], Nathan Cowieson[2] and Boyan Bonev[1#*]

**From the:**

[1]School of Life Sciences, University of Nottingham, Nottingham, NG7 2UH, UK

[2]Diamond Light Source, Didcot, Oxford, OX22 0DE, UK

[3]Nanoscale and Microscale Research Centre, University of Nottingham, Nottingham, NG7 2RD, UK

[4]School of Pharmacy, University of Nottingham, Nottingham, NG7 2RD, UK

[5]Mitsubishi Chemical Methacrylates, Wilton Centre, Wilton, Redcar, TS10 4RF UK

[#]University of Strasbourg Institute for Advanced Studies Fellow

[*]Corresponding author: boyan.bonev@nottingham.ac.uk

**Keywords**

Green chemistry, industrial biotechnology, sustainable manufacture, butyl methacrylate, membranes, lipid phases, solid state NMR, SAXS

**Abbreviations**

PMMA – polymethyl methacrylate; DOPC – C18Δ9-*cis* dioleoyl phosphatidyl choline; DEPC – C18Δ9-*trans* dipalmitoyl phosphatidyl choline; MO – monoolein; LCP – lipidic cubic phase; BMA – butyl methacrylate; NMR – nuclear magnetic resonance; ssNMR – solid state NMR; SAXS – small angle X-ray scattering; cryoEM – cryo-electron microscopy; FIB – focussed ion beam

## Experimental protocols

### Materials

All synthetic lipids were purchased from Avanti Polar Lipids (AL, USA) at >98% purity and used without further purification. Butyl methacrylate was supplied by Mitsubishi Chemical Corporation partner from Tokyo Chemical Industry at >99% purity and used without further treatments. Membrane models have been prepared from hydrated di-C18:1-Δ9-*cis* PC (DOPC, 1,2-dioleoyl-sn-glycero-3- phosphatidylcholine), or di-C18:1-Δ9-*trans* PC (DEPC, 1,2-dielaidoyl-sn-glycero-3-phosphatidylcholine) with effective hydrophobic thickness equivalent to C16:0 (27 Å) and C18:0 (30 Å), respectively, in the form of multilamellar liposome suspensions[1].

### LCP topology surfaces

Generating surfaces for the three lipid cubic phases in Figure 1 were created in Maple 2024 following[2]:

Primitive ***Im3m***:

sin(x).sin(y).sin(z) + sin(x).cos(y).cos(z) + cos(x).sin(y).sin(z) + cos(x).cos(y).sin(z) = 0

Gyroid $\boldsymbol{Ia\bar{3}d}$:

sin(x).cos(y)+sin(y).cos(z)+sin(z).cos(x) = 0

Diamond $\boldsymbol{Pn\bar{3}m}$:

cos(x) + cos(y) + cos(z) = 0

Cubic phase symmetries were assigned from reflections according to Table ST1.

Nanocrystallite size was estimated using the Scherrer formula:

$$D = \frac{K\lambda}{\beta.cos\theta}$$

Where K is the Scherrer constant, $\lambda$ is the X-ray wavelength, $\beta$ is the full width at half height of the reflection, and $\theta$ is the angle of reflection. For close to spherical nanocrystals, $K \approx 0.9$.

*Sample preparation*

Prior to BMA incorporation, 20 µmol lipids were hydrated in 1 ml of dd$H_2O$ for > 2 h to ensure formation of stable lipid bilayer. This was done above the lipid main transition temperature to ensure all lipid molecules self-assemble into membrane bilayers. The membrane was then centrifugated at 21382×g (Fisherbrand GT1R Centrifuge) where multilamellar vesicles (MLVs) were collected in the pellet and excess water was removed. BMA was added directly to the MLV pellets and stirred well with a fine glass rod. BMA was added to the lipid system as lipid-to-BMA molar ratios of 2:3, 1:3, 1:6 and 1:9. Hydration levels were monitored at every stage by following the sample weight.

*Solid state NMR*

Solid state NMR experiments were performed on Varian 400 MHz VNMRS spectrometer equipped with a 4 mm MAS NMR probe. Temperature was regulated using balanced heated/vortex tube-cooled gas flow, and the measured values were corrected for known heating due to MAS and RF[3,4]. All $^{31}P$ spectra were referenced externally to 85% $H_3PO_4$ at 0 ppm. Wideline $^{31}P$ ssNMR was carried out at a frequency of 161.82 MHz at 20 °C, using the Hahn echo sequence with 100 kHz $\pi/2$ and $\pi$-pulses separated by 12 µs interpulse and pre-acquisition delays. Spectra were acquired with 50 ms acquisition time with a recycle delay of 5 s and 1024 transients were averaged to obtain each FID. All spectra were processed with 100 Hz Lorentzian line broadening.

*Small angle X-ray scattering, SAXS*

Samples were transferred onto multipurpose sample cells by sandwiching between two thin Kapton polyimide films, on a 1.6 mm resin 3D printed stick and secured using Kapton tapes[5]. SAXS 2D datasets were recorded at 20˚C using a Dectris EigerX 4M with a Q range of 0.0045-0.34 Å$^{-1}$, Energy 13.1 keV and a sample to detector distance of 3688 mm. Data was analysed using Scatter 3 (Diamond Light Source, UK).

<u>FIB-SEM</u>

CryoTEM samples were frozen on copper TEM finder-grids using a Gatan CP3 (Pleansanton CA, USA) or a Leica EM GP2 (Wetzlar, Germany) ethane cryoplunge system, without blotting the sample. For DOPC/BMA 1:6, after freezing they were secured to the cryo-SEM shuttle (Zeiss cryo-correlative stage at 45 degree pre-tilt) and the cryo-sledge with the sample was transferred to the preparation chamber. Sample was sputter coated with Pt at a current of 10 mA for 60 s.

Cryo-SEM was performed using a Zeiss Crossbeam 550 (Carl Zeiss, Oberkochen, Germany) focused ion beam scanning electron microscope (FIB-SEM) at an accelerating voltage of 2 kV. The samples were maintained at cryogenic temperatures of -170°C using a Quorum 3010 Cryo stage (Quorum Technologies, Loughton, UK). Samples were transferred to the main stage of the microscope and imaged using a range of detection modes including the SESI and inLens detectors at 2 kV accelerating voltage. Gallium Ion column conditions were 30 kV and currents between 50 pA and 7 nA. Prior to the Cryo-FIB thinning, a layer of organometallic precursor was deposited from the gas injector system (GIS) for approximately 45-60 s.

<u>*Electron microscopy*</u>

Sample were prepared on copper TEM grid, as describe aboved. Once vitrified, samples were maintained under liquid nitrogen until transfer to the cryoTEM sample holder (Gatan 626 cryoholder and Smartset controller) for TEM imaging at 170°C. Cryo-TEM was performed at an

operating voltage of 200 kV, with nominal defocus, on either a JEOL 2100F (Tokyo, Japan) equipped with a Gatan K3-IS direct detection camera, or on a JEOL 2100Plus equipped with a Gatan OneView CMOS camera.

**Supplemental figures**

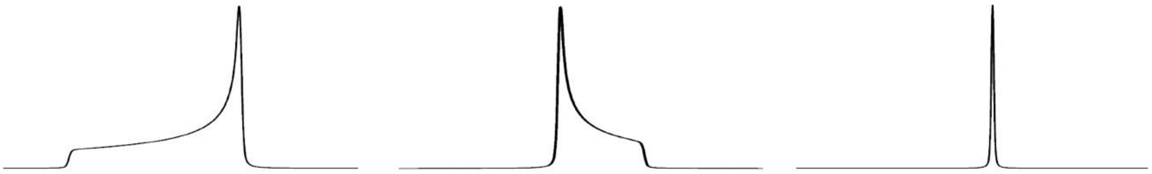


**Figure S1**. Theoretical wideline $^{31}$P NMR intensity distributions, used to identify bilayers, lamellar phase $\boldsymbol{L_\alpha}$, inverted hexagonal, $\boldsymbol{H_{II}}$, and lipidic cubic phases, **Ia3d**, **Pn3m** and **Im3m**.

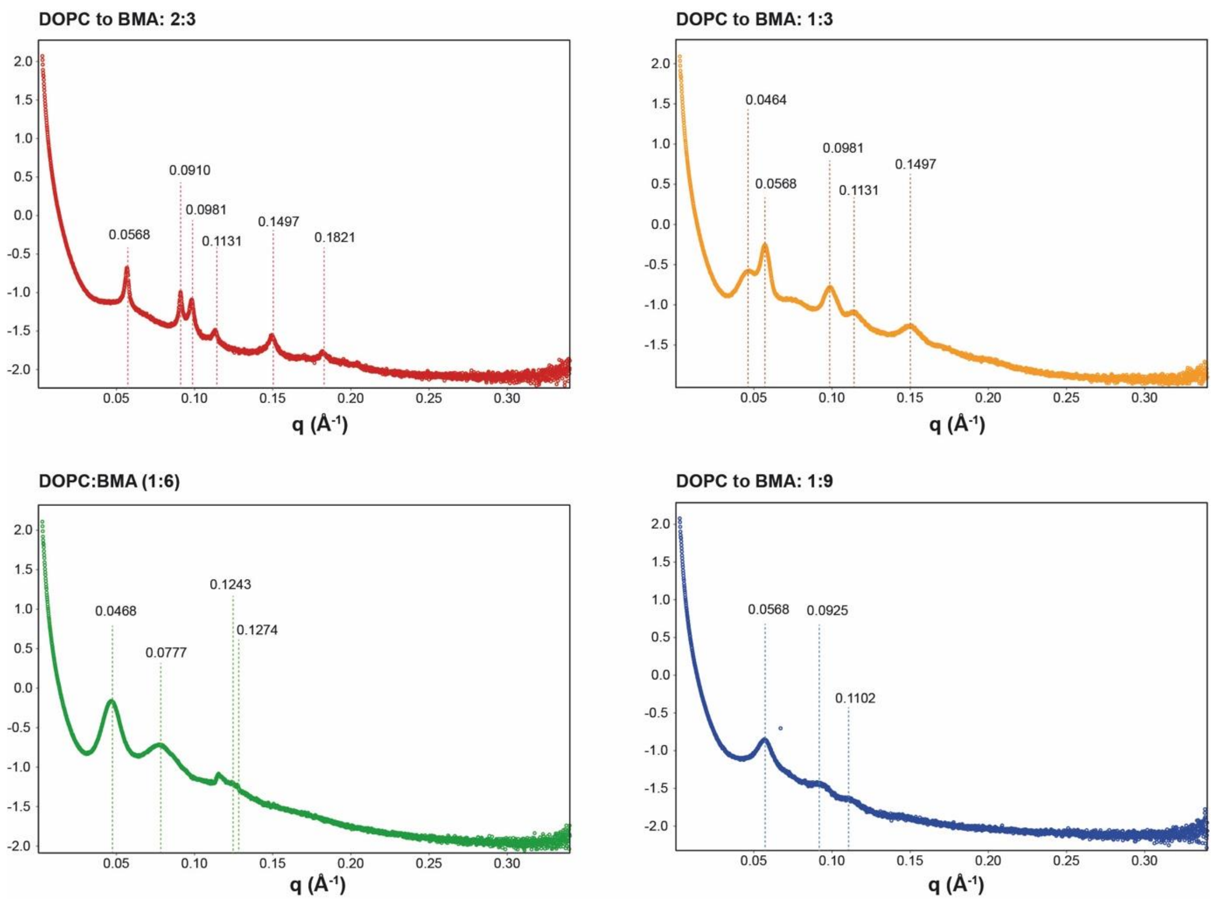


**Figure S2**. 1D SAXS Debye-Scherrer powder diffraction patterns from hydrated DOPC lipids with different concentration of BMA at molar ratios of 2:3 (red); 1:3 (yellow); 1:6 (green) and 1:9 (blue) lipid to BMA

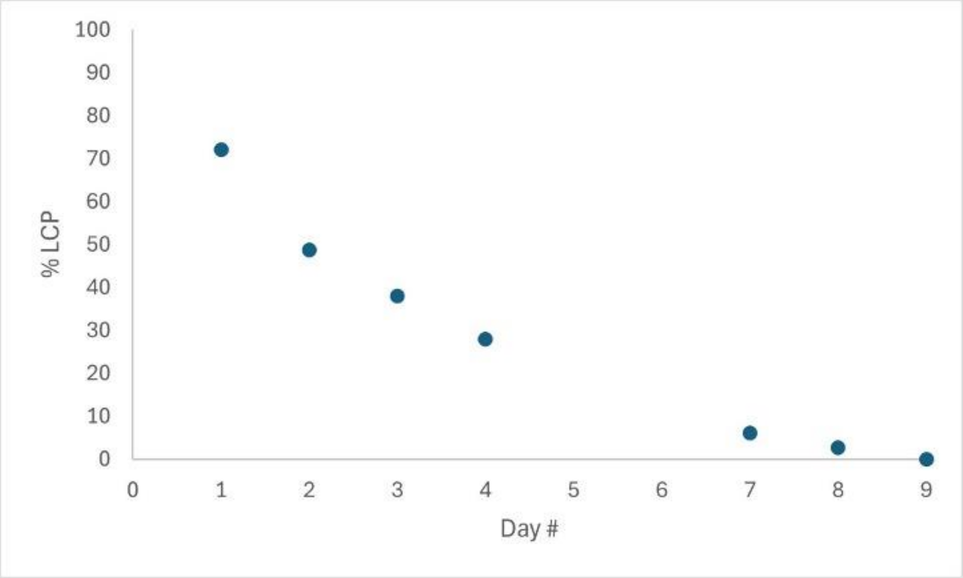


**Figure S3**. Percentage of cubic phase population of DEPC/BMA with 1:6 molar ratio with respect to time, measured from $^{31}$P NMR spectra shown in Figure 4. Spectra were not recorded on Days 5 and 6.

**Lipid phase identification from wideline $^{31}P$ NMR spectra and X-ray diffraction patterns**

Maintaining membrane integrity is crucial to cellular survival and the latter outcome is a pivotal determinant of a successful strain development for biofermentative production of industrial solvents[6]. Solvent-induced instabilities lead to loss of membrane integrity, non-bilayer phase formation and collapse of transmembrane electrochemical and solute gradients. Lipid phase analysis is done by ssNMR. The bilayer phase gives rise to a powder distribution, which is recognised in a 90˚ edge at -16 ppm. Inverted powder distribution, characteristic of the $\mathbf{H_{II}}$ phase, is resolved from the 90˚ edge at 8 ppm. A small fractional contribution (around 4%) from a distended LCP is seen as an isotropic spectral feature near -1 ppm (Figure S1).

Further classification and structural characterisation of LCPs is done using Debye-Scherrer powder diffraction under SAXS conditions. Phase coexistence is analysed using a sliding "Vernier"-like scale, using reflection patters described in ST1.

| Q-ratios | Ia3d | Pn3m | Im3m | H | L |
|---|---|---|---|---|---|
| 1 | x | x | x | x | x |
| 1.15 | x | | | | |
| 1.22 | | x | | | |
| 1.41 | | x | x | | |
| 1.53 | x | | | | |
| 1.63 | x | | | | |
| 1.73 | | x | | x | |
| 1.83 | x | | | | |
| 1.91 | x | | | | |
| 2 | | x | x | x | x |
| 2.12 | | x | | | |
| 2.24 | | | x | | |
| 2.45 | | | x | x | |

**Table ST1**: **Characteristic XRD patters used in phase identification**[7]. Mixed phases were assigned by applying this stencil sequentially from the lowest to higher Q reflections and matching the reflections to tabulated patterns.

## Phase analysis from SAXS powder diffraction

*Phase analysis of DOPC/BMA/water ternary systems*

Debye-Scherrer powder diffraction under SAXS from 2:3 DOPC/BMA hydrated systems reveals the [100] n = 1 and n = 2 lamellar reflections at q = 0.0910 $Å^{-1}$ and 0.182 $Å^{-1}$, respectively, correspond to a lamellar repeat ***d*** of 69.0 Å, slightly swollen compared to pure hydrated DOPC repeat of 62.8 Å[1]. The $\mathbf{H_{II}}$ phase gives rise to a [110] reflections at q = 0.0568 $Å^{-1}$, as well as a characteristic reflection at √3 = 1.73 with q = 0.0981 $Å^{-1}$ correspond to $d_{110}$ = 111 Å and unit cell dimension a = 2.$d_{110}$/√3 = 128 Å  (Table ST1). The presence of a distended LCP with **Ia3d** symmetry was identified from the Bragg reflections correspond to √4:√3 = 1.15 and q = 0.113 $Å^{-1}$, and √7:√3 = 1.53 with q = 0.150 $Å^{-1}$. These parameters indicate a unit cell size a = 64.1 Å. Considering the Bragg reflections width and using the Scherrer formula, we estimate the size of ordered structures, giving rise to the Bragg reflections, to be on the order of 4200 Å.

Diffraction patterns observed at 1:3 lipid-BMA are characterised by Bragg reflections belonging both to LCP with **Pn3m** symmetry giving rise to reflections at q = 0.046 $Å^{-1}$, √4:√3 = 2.15 and q = 0.0740 $Å^{-1}$, which correspond to a unit cell with dimension a = 135 Å. Coexisting with the **Pn3m** LCP, we observed an **Ia3d** phase, as observed at the lower BMA ratio at q = 0.981 $Å^{-1}$ with unit cell size a = 64 Å. Reflections at √3:√2 = 1.22 with q = 0.0568 $Å^{-1}$, and √9:√2 = 2.12 with q = 0.0981 $Å^{-1}$, overlap with the $\mathbf{H_{II}}$ and the **Ia3d**, respectively.

At 1:6 DOPC/BMA, the $^{31}$P wideline NMR 90-degrees edge at 8 ppm, characteristic of a $\mathbf{H_{II}}$ phase, disappeared from the spectra and the q = 0.0568 $Å^{-1}$ Bragg reflection was also absent from the 1D SAXS profile. While we could not resolve coexisting cubic phase symmetries from the $^{31}$P wideline

NMR spectra, the Bragg reflection at q = 0.0468 $Å^{-1}$ indicates the presence of a **Pn3m** cubic phase with characteristic dimension ***a*** = 134 Å, as observed in 1:3 DOPC/BMA systems. In addition, the reflections at q = 0.0777 $Å^{-1}$, √7:√3 = 1.53 with q = 0.1243 $Å^{-1}$ and √8:√3 = 0.163 with q = 0.1274 $Å^{-1}$ indicate the presence of an **Ia3d** phase. Using the Scherrer equation with the lowest q Bragg reflection width, we estimate the size of the diffracting periodic structures, cubosomes[8], to be small and on the order of 900 Å.

At the highest BMA concentration of 1:9 DOPC/BMA, $^{31}P$ wideline NMR shows a single cubic phase (Figure 2F). SAXS profiles reveal Bragg reflections shifted to higher q, which are challenging to interpret unambiguously. The reflection at q = 0.0468 $Å^{-1}$ form the **Pn3m** LCP was absent but a reflection at q = 0.0568 $Å^{-1}$, attributable to an $\mathbf{H_{II}}$ phase, was observed, instead (Figure 2H). Yet, the $\mathbf{H_{II}}$ signature √9:√3 = 1.73 reflection is absent. Considered together, the reflections at q = 0.0925, √8:√6 = 0.115 and q = 0.110 $Å^{-1}$, and √8:√3 = 0.163 and q = 0.145 $Å^{-1}$, suggest the presence of an **Ia3d** cubic phase. These observations, together with the broad Bragg reflections, may be accounted for by considering a distorted phase, as reported in some monoolein-based LCPs[7].

*Phase behaviour of DOPC/BMA/water ternary systems*

Diffraction patterns from hydrated 1:6 DEPC/BMA systems show Bragg reflections with n = 1 at q = 0.043 $Å^{-1}$ and q = 0.059 $Å^{-1}$, which we attribute to coexistence of **Pn3m** and **Ia3d** phases with ***a*** = 146 Å and 107 Å, respectively (Figure 3B). The size of diffracting nanocrystallites was estimated using the Scherrer formula form Bragg reflection widths to be on the order of 80 nm. In addition to LCPs, Bragg reflections in 1D SAXS profiles reveal coexistence of a residual bilayer component at n = 1 and n = 2 with q = 0.102 $Å^{-1}$ and q = 0.203 $Å^{-1}$, respectively. These Bragg reflections reveal lamellar repeat d = 61.6 Å, slightly lower than the lamellar repeat of 64.1 Å observed in hydrated

DEPC membrane. This is expected as a result from increased lipid chain disorder in the presence of solvent.

The phase coexistence in hydrated 1:6 DEPC/BMA systems give rise to a single isotropic feature in the $^{31}$P NMR spectra in Figure 3. Besides LCP, isotropic wideline $^{31}$P NMR spectra can arise from small, rapidly tumbling aggregates in solution[9,10]. In this study, the samples diffract with reflection patterns corresponding to the presence of LCP and the $^{31}$P NMR linewidth is greater than the typical narrow lines, observed for free tumbling structures[9,10]. Also, the sample appearance has toothpaste-like consistency in-keeping with known LCP viscosity, unlike free tumbling small structures in low viscosity suspensions. All that said, we cannot rule out entirely the presence of free tumbling small aggregates from SAXS and $^{31}$P NMR data. In a three-component system with three coexistent phases, such additional phase limits thermodynamic degrees of freedom and can only occur over a restricted area of the thermodynamic space.

**Discussion**

Membrane phase behaviour and stability of hydrated lipid/BMA mesophases are critical determinants of cellular stability in the presence of solvent and depend on lipid chain composition[1]. In turn, this is determined by the molecular architecture of membrane lipids, which can absorb or expel hydrophobic solvents with resulting phase polymorphism. Unlike hydrated MO systems, in which lamellar phases are not adjacent to **HII** phases but are separated by LCPs[11], ternary systems of hydrated lipid/BMA mixtures reveal adjacency of lamellar, **HII** and LCPs. The presence of **Pn3m/$H_{II}$** phase boundary we observe for phospholipid/BMA systems in this study has also been discussed in hydrated monoolein mixed mesophases[12].

We illustrate a solvent driven phase conversion from membrane via $H_{II}$ to LCP pathway in solvent-supersaturated hydrated lipid systems, which can be reversed in *trans*-unsaturated C18:1 chain lipids to recover the stable membrane phase. In sustainable manufacture of commodity chemicals, the use of engineered fermentation organisms with *trans*-C18:1 lipid can support membranes at very high levels of solvent, while having the ability to restructure and expel excessive product through bilayer phase regeneration. With *cis*-C18:1 unsaturated chain lipids, the LCPs remain stable with solvent-saturated hydrophobic interior. This offers a new dimension for the development of 3D nanomaterials using a hydrophobic solvent.

REFERENCES


(1) Yeh, V.; Goode, A.; Eastham, G.; Rambo, R. P.; Inoue, K.; Doutch, J.; Bonev, B. B. Membrane Stability in the Presence of Methacrylate Esters. *Langmuir* **2020**, *36* (33), 9649–9657. https://doi.org/10.1021/acs.langmuir.9b03759.

(2) Rummel, G.; Hardmeyer, A.; Widmer, C.; Chiu, M. L.; Nollert, P.; Locher, K. P.; Pedruzzi, I.; Landau, E. M.; Rosenbusch, J. P. Lipidic Cubic Phases: New Matrices for the Three-Dimensional Crystallization of Membrane Proteins. *Journal of Structural Biology* **1998**, *121* (2), 82–91. https://doi.org/10.1006/jsbi.1997.3952.

(3) Ciesielski, F.; Griffin, D. C.; Rittig, M.; Bonev, B. B. High-Resolution J-Coupled 13C MAS NMR Spectroscopy of Lipid Membranes. *Chemistry and Physics of Lipids* **2009**, *161* (2), 77–85. https://doi.org/10.1016/j.chemphyslip.2009.07.001.

(4) Zorin, V.; Ciesielski, F.; Griffin, D. C.; Rittig, M.; Bonev, B. B. Heteronuclear Chemical Shift Correlation and J-Resolved MAS NMR Spectroscopy of Lipid Membranes. *Magnetic Resonance in Chemistry* **2010**, *48* (12), 925–934. https://doi.org/10.1002/mrc.2690.

(5) Edwards-Gayle, C. J. C.; Khunti, N.; Hamley, I. W.; Inoue, K.; Cowieson, N.; Rambo, R. Design of a Multipurpose Sample Cell Holder for the Diamond Light Source High-Throughput SAXS Beamline B21. *J Synchrotron Rad* **2021**, *28* (1), 318–321. https://doi.org/10.1107/S1600577520013831.

(6) Webb, J. P.; Paiva, A. C.; Rossoni, L.; Alstrom-Moore, A.; Springthorpe, V.; Vaud, S.; Yeh, V.; Minde, D.-P.; Langer, S.; Walker, H.; Hounslow, A.; Nielsen, D. R.; Larson, T.; Lilley, K.; Stephens, G.; Thomas, G. H.; Bonev, B. B.; Kelly, D. J.; Conradie, A.; Green, J. Multi-Omic Based Production Strain Improvement (MOBpsi) for Bio-Manufacturing of Toxic Chemicals. *Metabolic Engineering* **2022**, *72*, 133–149. https://doi.org/10.1016/j.ymben.2022.03.004.

(7) Joseph, J. S.; Liu, W.; Kunken, J.; Weiss, T. M.; Tsuruta, H.; Cherezov, V. Characterization of Lipid Matrices for Membrane Protein Crystallization by High-Throughput Small Angle X-Ray Scattering. *Methods* **2011**, *55* (4), 342–349. https://doi.org/10.1016/j.ymeth.2011.08.013.

(8) Demurtas, D.; Guichard, P.; Martiel, I.; Mezzenga, R.; Hébert, C.; Sagalowicz, L. Direct Visualization of Dispersed Lipid Bicontinuous Cubic Phases by Cryo-Electron Tomography. *Nat Commun* **2015**, *6* (1), 8915. https://doi.org/10.1038/ncomms9915.

(9) Bonev, B. B.; Lam, Y.-H.; Anderluh, G.; Watts, A.; Norton, R. S.; Separovic, F. Effects of the Eukaryotic Pore-Forming Cytolysin Equinatoxin II on Lipid Membranes and the Role of Sphingomyelin. *Biophysical Journal* **2003**, *84* (4), 2382–2392. https://doi.org/10.1016/S0006-3495(03)75044-9.

(10) Bonev, B. B.; Gilbert, R. J. C.; Andrew, P. W.; Byron, O.; Watts, A. Structural Analysis of the Protein/Lipid Complexes Associated with Pore Formation by the Bacterial Toxin Pneumolysin. *J. Biol. Chem.* **2001**, *276* (8), 5714–5719. https://doi.org/10.1074/jbc.M005126200.

(11) Qiu, H.; Caffrey, M. The Phase Diagram of the Monoolein/Water System: Metastability and Equilibrium Aspects. *Biomaterials* **2000**, *21* (3), 223–234. https://doi.org/10.1016/S0142-9612(99)00126-X.

(12) Yao, Y.; Catalini, S.; Foggi, P.; Mezzenga, R. Water–Lipid Interface in Lipidic Mesophases with Excess Water. *Faraday Discuss.* **2024**, *249* (0), 469–484. https://doi.org/10.1039/D3FD00118K.
